# Teledermatology in Norway using a mobile phone app

**DOI:** 10.1371/journal.pone.0232131

**Published:** 2020-04-27

**Authors:** Syed Mohammad Husain Rizvi, Thomas Schopf, Amandip Sangha, Kim Ulvin, Petter Gjersvik

**Affiliations:** 1 Department of Dermatology, Oslo University Hospital, Oslo, Norway; 2 Askin AS, Oslo, Norway; 3 National Centre for e-Health Research, University Hospital Northern Norway, Tromsø, Norway; 4 Institute of Clinical Medicine, University of Oslo, Oslo, Norway; Leiden University Medical Center, NETHERLANDS

## Abstract

Rashes, ulcers and skin lesions are well suited for telemedicine. We have developed a smartphone app, the first of its kind in Norway, where a referring physician can write a short medical history and take clinical and dermatoscopic photographs with a smartphone, which is then sent to and evaluated by a dermatologist. In the period from June 1^st^, 2017, to September 1^st^, 2019, clinical information and photographs of rash and skin lesions from 171 patients were sent by 40 primary care and nursing home physicians via the smartphone app to four dermatologists for diagnosis and therapeutic advice. A wide range of dermatological conditions were diagnosed, most commonly chronic ulcers (17%), eczema (15%) and pigmented lesions (13%). Assessed later by a dermatologist, referral for regular consultations with a specialist was avoided in 119 patients (70%). Sixteen patients (9%) were recommended a regular consultation with a dermatologist; information for prioritization in the specialist healthcare service was then provided. In 36 patients (21%), further measures by the referring physician were recommended. Our experience indicates that many ordinary consultations on rash, ulcers and skin lesions in the specialist healthcare services can be avoided when using the smartphone app.

## Introduction

Teledermatology is the use of telemedicine for the diagnosis and treatment of rash, ulcers and skin lesions [1–3]. Several studies have shown that teledermatology is a good alternative to regular consultations with a dermatologist [4–6]. Most patients with skin problems are treated by primary care physicians, but many patients need the assessment of a specialist. Due to the limited number of dermatologists in Norway, patients’ waiting time for a specialist consultation is often long [7].

We have developed an app where the referring physician with their smartphone can type a brief medical history and take clinical and dermatoscopic photographs, which are then sent to and evaluated by a dermatologist. The project is the first of its kind in Norway and seeks to build a professional and economically sustainable digitalized skin care service. We describe here our experiences from the pilot phase of the project.

## Material and methods

**Fig 1:**
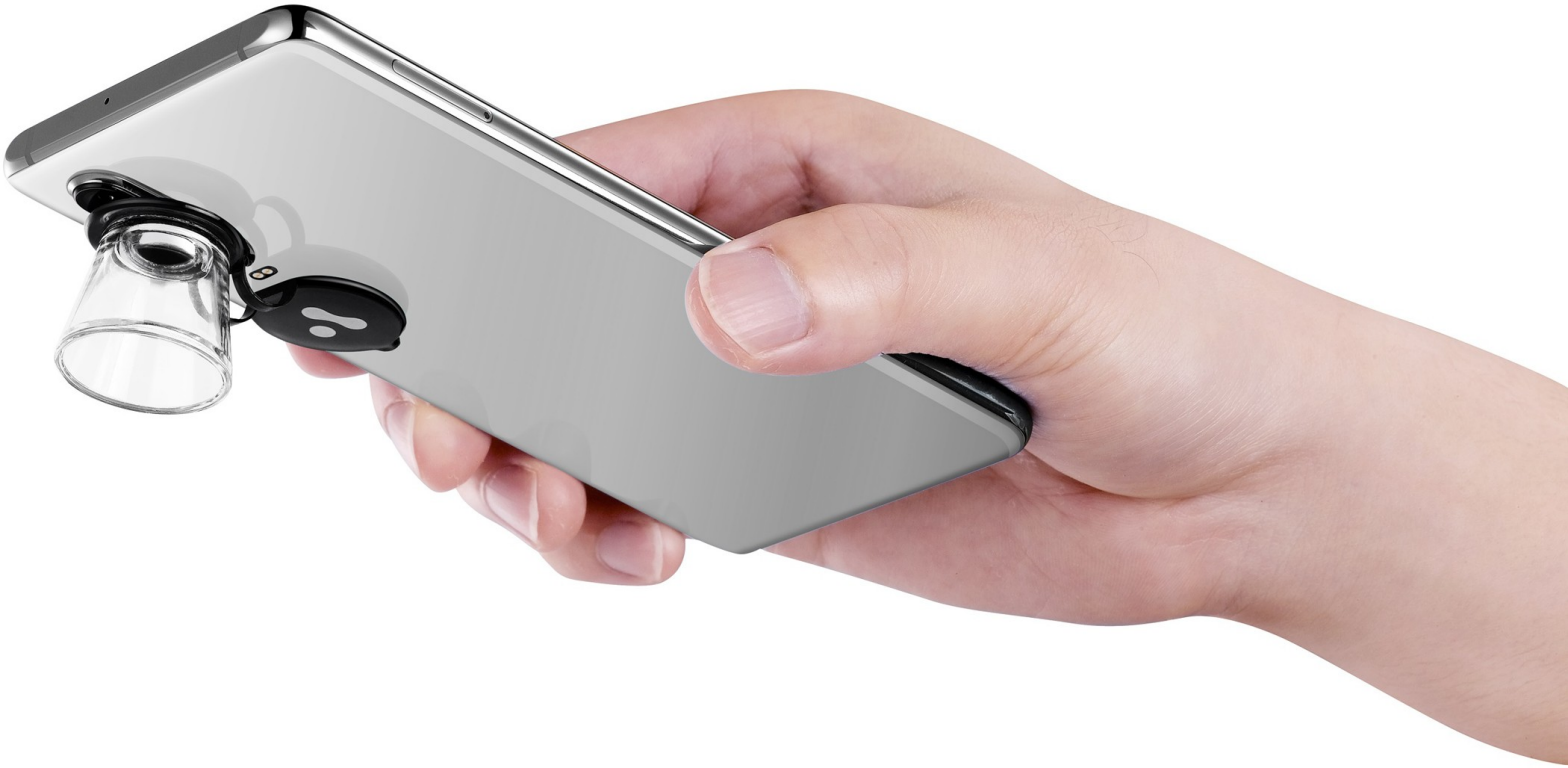
The *AskinScope®* dermatoscopy lens.

The app *Askin®* was downloaded from the Apple App store or Google Play store on the referring physician's smartphone. The referring physician provided a brief history, answered 1–5 predefined questions and took a photograph of the skin area in question. For pigmented skin lesions and other skin lesions, it was recommended to submit dermatoscopic images using the *AskinScope®* dermatoscopy lens, a newly developed dermatoscope magnification lens that can be attached to the smartphone's camera (Fig 1). Referrals submitted were reviewed by one of four dermatologists, and referring physicians received responses via the same app.

For this report, the diagnoses were categorized into the following diagnostic categories by the dermatologists: eczema, pigmented skin lesions, benign tumours, skin cancer, skin infections, chronic ulcers, other diagnoses (i.e. diagnoses that did not fit into the above categories), and uncertain diagnosis (i.e. when a clear diagnosis was not possible and several differential diagnoses were suggested). All the diagnoses were based on the patient history and the images sent by the referring doctor. In some cases, additional information about the patient’s conditions was obtained. All consultations were analysed after the pilot period by a dermatologist to evaluate whether using the app meant that referral for a regular consultation had been avoided. It was also recorded whether referral to regular consultations with a dermatologist or further action by referring physician was recommended.

The norm for information security and privacy in the health and care service in Norway was followed [8]. The study was submitted to the South-East Regional Committee for Medical and Health Research Ethics (ref.no.:2017/834C), which considered the study to be a quality assurance study without the need for ethical approval. The consent of sending a consultation was given verbally to the referring doctor. In case of minors, the consent was given by the parents. In case of elderly patients with reduced cognitive function/ dementia, the consent was given by the family members, or the referring doctor had the authority from the family members.

## Results

Forty primary care physicians and nursing home were registered as users in the pilot phase from June 15^th^, 2017 to August 15^th^, 2019. In total, 178 referrals were submitted. Three referrals were excluded because the referring physician had duplicated the referral and four due to technical problems; 171 referrals were thus included in the analysis.

The average response time was 9 hours. The most frequent diagnostic categories were chronic ulcers (n = 29) and eczema (n = 23) (Table 1). Twenty-two referrals were for pigmented skin lesions, and over half of these patients needed no further action. Malignant melanoma was diagnosed in two patients.

**Table 1:** Overview of 171 teledermatological consultations.

Women / men (n; %)	109/62	(64/36)
Age (years; median; spread)	77	(1–103)
Duration of skin problem (n; %)		
> 1 year	46	-27
2–12 months	64	-37
2–8 weeks	31	-18
<14 days	30	-18
Response time (hours; mean; spread)	9	(1–30)
Diagnostic categories (n;%):		
Chronic ulcers	29	-17
Eczema	23	-13
Pigmented skin lesions	22	-13
Skin cancer	21	-12
Benign tumours	16	-9
Skin infections	16	-9
Other diagnoses	30	-18
Uncertain diagnosis	14	-8

In all, 119 patients (70%) were considered not to need a regular dermatologist consultation after being assessed via the app. Sixteen patients (9%) were recommended to seek a dermatologist for a regular consultation and were given a written referral with clinical information so that patients could be prioritized more easily in the specialist health service. Finally, 36 patients (21%) were recommended further measures by the referring physician, including biopsy, curettage, excision, topical treatment and wound procedures.

## Discussion

In this pilot study on the use of a smartphone app for specialist assessment of rash and skin lesions, 70% of patients were considered to have avoided regular consultation in the specialist health service. These experiences correspond to similar studies in other countries. In a large study from the Netherlands with 37,207 teledermatological consultations after referral from 1,820 general practitioners, 74% of patients were considered to have avoided referral to regular dermatology consultations [6]. A smaller study from Belgium found that the number of such referrals was reduced by 71% [9]. Avoiding referral for regular consultations in the specialist health service entails major financial, organizational and logistical benefits, and patients avoid unnecessary suffering and waiting time.

The consultations covered a wide range of diagnoses, with chronic ulcers being the most frequent diagnosis category. The dermatologist's most important task was to optimize the wound treatment, but often also to provide suggestions for diagnostic assessment and follow-up. Chronic ulcers are common among the elderly, especially in nursing homes. A study suggested that patients with chronic ulcers can be treated to a greater extent in primary health care using telemedicine technology [10].

About one-third of the consultations involved pigmented skin lesions and tumours. Our experience suggests that teledermatology can be part of a triage system where patients with cancer-suspect skin lesions can be referred more quickly to the specialist health service and be prioritized there according to the set priorities. A study from Spain found that more than half of all patients referred for a skin tumour assessment did not really need a regular dermatologist consultation [11]. In a Swedish study, the use of smartphone teledermatoscopy referrals resulted in a faster and more efficient management of skin cancer patients as compared to traditional paper referrals [12]. Limitations to this study includes absence of a physical consultation by a dermatologist to confirm the diagnosis and follow-up data on clinical and management outcome.

The use of teledermatological technology can result in reduced patient waiting time, faster start-up of treatment, reduced travel time, reduced health care costs and better access to specialist expertise. It can also be a good learning arena for the doctors involved. We want to try out our smartphone app in hospitals with and without dermatologists and in home-based nursing care, occupational health care and prison health care. We believe that smartphone-based teledermatological technology has a great potential, both in Norway and elsewhere.
